# Robust Line Segment Matching for Space-Based Stereo Vision via Multi-Constraint Global Optimization

**DOI:** 10.3390/s25175466

**Published:** 2025-09-03

**Authors:** Xingxing Zhang, Ling Wang

**Affiliations:** Key Laboratory of Radar Imaging and Microwave Photonics, Ministry of Education, Nanjing University of Aeronautics & Astronautics, Nanjing 210016, China; xxzhang_sd@163.com

**Keywords:** stereo vision, line segment matching, geometric constraints, cost matrix construction, Hungarian algorithm, space-based vision

## Abstract

Robust and accurate line segment matching remains a critical challenge in stereo vision, particularly in space-based applications where weak texture, structural symmetry, and strong illumination variations are common. This paper presents a multi-constraint progressive matching framework that integrates epipolar geometry, coplanarity verification, local homography, angular consistency, and distance-ratio invariance to establish reliable line correspondences. A unified cost matrix is constructed by quantitatively encoding these geometric residuals, enabling comprehensive candidate evaluation. To ensure global consistency and suppress mismatches, the final assignment is optimized using a Hungarian algorithm under one-to-one matching constraints. Extensive experiments on a wide range of stereo image pairs demonstrate that the proposed method consistently outperforms several advanced conventional approaches in terms of accuracy, robustness, and computational efficiency, as evidenced by both quantitative and qualitative evaluations.

## 1. Introduction

Accurate relative pose estimation of space targets is a fundamental prerequisite for enabling autonomous on-orbit servicing missions. Most artificial space targets are primarily composed of planar surfaces and linear edges (e.g., solar panel frames, satellite launch ring interfaces, and rectangular facets of cube satellites). Compared with other geometric primitives such as points or surfaces, line segments are less sensitive to illumination changes and sensor noise [1]. Therefore, representing space targets using line features can effectively suppress environmental interference in space and enhance the robustness of pose estimation algorithms.

Line segment matching is a fundamental task in computer vision and image processing, with applications not only in pose estimation [2,3] but also in target tracking [4,5], image stitching [6,7], and 3D reconstruction [8,9,10]. Beyond these application scenarios, advances in broader image processing tasks also provide relevant inspiration. For instance, underwater image enhancement often relies on correcting color disparities to achieve natural color consistency [11]. In recent years, extensive studies have been conducted on line segment matching. Existing approaches to line segment matching can be categorized into four classes [12]: (1) single-line-based methods, (2) line-group-based methods, (3) coplanar line-point invariant methods, and (4) deep learning-based methods.

Single-line-based methods are typically statistical approaches that describe local affine-invariant features within the support region of a line segment [13]. Bay et al. [14] first constructed line descriptors using color histograms and enhanced matching accuracy by incorporating topological filtering. Wang et al. [15] proposed the classic mean-standard deviation line descriptor (MSLD), which partitions a parallel neighborhood of the line into subregions and computes directional gradient histograms. These histograms are then summarized using statistical descriptors to improve robustness. However, MSLD may suffer under complex textures and rotational variations. Wang et al. [16] further introduced a descriptor based on rotation-invariant Harris features derived from image gradients. To address scale sensitivity, Zhang et al. [17] developed the line band descriptor (LBD), which constructs descriptors across image pyramids and utilizes stripe-based subregions along the line direction. Liu et al. [18] extended point-based descriptors to construct global descriptors for lines and curves using edge points. More recently, Lin et al. [19] proposed the illumination-insensitive line binary (IILB) descriptor, which is robust to severe illumination variations through hierarchical band differencing. Zhang et al. [20] introduced dual point–line invariants that jointly exploit distance-ratio and directional-vector constraints, thereby enhancing robustness in low-texture scenarios where conventional descriptors often fail.

Line-group-based methods enhance matching reliability by incorporating structural or geometric constraints among neighboring lines. Schmid et al. [21] modeled inter-line relationships using graphs for planar scenes. Al-Shahri et al. [22] leveraged epipolar and coplanar constraints to filter matches. Ok et al. [23] used seven geometric constraints for aerial stereo image matching. López et al. [24] integrated appearance, geometry, and contextual information, achieving robust performance in low-texture scenarios. Kim et al. [25] proposed a method tailored for low-texture images using intersection points of coplanar lines as canonical patches. Following this, various techniques exploiting the invariance of such intersections have been proposed. Li et al. [26] introduced the line-junction-line (LJL) algorithm, which constructs gradient descriptors around junctions and refines matches through propagation and local homography verification. An improved version was proposed in [27]. Chen et al. [28] also extended the LJL algorithm. Zheng et al. [29] presented the smoothly varying projective transformation for line segment matching (SLEM), which builds global and smooth projective models from point matches for line validation, though the approach is sensitive to point matching quality and is computationally expensive. Scavello et al. [30] proposed a descriptor encoding relative positions of nearby lines without relying on appearance. More recently, Wei et al. [31] proposed the graph-based line segment matching (GLSM) algorithm, which constructs a line-point graph based on geometric, epipolar, and trifocal constraints and applies a reweighted random walk (RRWM) algorithm for global optimization, particularly suited for stereo and multi-view matching.

Coplanar line-point invariant methods utilize projection-invariant geometric structures composed of lines and coplanar points. Fan et al. [32,33] proposed to compute line-point invariants from the line’s adjacent regions and match line segments using maximum similarity of invariant values. Jia et al. [34] extended this concept with a novel five-point invariant based on the characteristic number (CN) [35], which exhibits strong performance under wide baselines and low-texture conditions, though limited to planar configurations.

With the rapid development of deep learning, line segment matching has increasingly shifted from handcrafted geometric heuristics toward data-driven frameworks that enable more robust feature representation learning and even end-to-end correspondence optimization. Early studies, such as the work of Vakhitov et al. [36], who trained a modified L2-Net descriptor with triplet loss, laid the foundation for subsequent learning-based approaches. Building on this direction, Ma et al. [37] proposed a graph convolution network (GCN) framework that jointly learns descriptors and establishes correspondences by formulating line segment matching as an optimal transport problem, yielding substantial improvements in recall compared with traditional methods. Li et al. [38] further advanced this paradigm by introducing a neural network framework to verify candidate line correspondences, which outperformed purely geometric selection strategies. More recently, structural representation models such as GlueStick [39] have exploited graph neural networks to perform joint point–line segment matching on wireframe structures, thereby providing robust performance under challenging viewpoint and illumination variations. In parallel, representative systems such as the self-supervised occlusion-aware line description and detection (SOLD2) [40] have demonstrated the feasibility of self-supervised learning by integrating detection and description within a single network, thus producing reusable line descriptors for correspondence tasks. Building on this direction, Abdellali et al. [41] developed a self-supervised framework that learns line descriptors from normalized regions of support without manual annotations, while Yoon et al. [42] proposed a context-aware descriptor incorporating attention mechanisms to emphasize discriminative points along a line, thereby improving robustness under noise and partial occlusion. Despite these advances, their applicability in on-orbit space scenarios remains limited due to computational constraints and the scarcity of annotated spaceborne data.

Despite substantial progress in line segment matching, most existing algorithms are developed for generic vision tasks and fall short of meeting the stringent requirements of on-orbit servicing. Two mission-critical challenges remain unresolved. First, the extreme and dynamically varying illumination in space frequently causes inconsistent or missing line detections across stereo views, and such errors propagate through the matching stage, ultimately compromising pose estimation. Second, the limited computational resources available onboard satellites impose strict efficiency constraints, making solutions that are both lightweight and real-time essential. These challenges materially affect the reliability of line segment matching and, consequently, the guidance and control of non-cooperative space missions. Motivated by these deficiencies, this study introduces a semi-physical simulation platform to provide representative testing conditions, employs the space target line segment detector (ST_LSD) [43] to improve line extraction robustness under adverse illumination, and develops a multi-constraint global optimization framework that enhances matching reliability while maintaining computational efficiency. Importantly, the proposed constraints function as lightweight geometric checks that progressively eliminate implausible correspondences, thereby reducing the effective search space and enabling more efficient assignment.

In this work, we introduce a novel multi-constraint line segment matching framework specifically designed for stereo vision-based pose estimation of non-cooperative space targets. Building upon the unified geometric constraint formulation of GLSM, our method integrates several tailored innovations. We adopt the ST_LSD detector to ensure robust line segment extraction in spaceborne imaging scenarios with weak texture and unstable illumination. Crucially, instead of relying on an assumed coplanarity, we leverage 3D geometric cues obtained via stereo triangulation to verify coplanarity explicitly. A comprehensive cost matrix is constructed by jointly incorporating multiple geometric and structural constraints, including epipolar consistency, coplanarity validation, local homography compatibility, angular alignment, and distance-ratio invariance. The final assignment is solved with an enhanced Hungarian algorithm, which efficiently suppresses high-cost outliers while maintaining global optimality.

The remainder of this paper is organized as follows. Section 2 provides an overview of the proposed method. Section 3 introduces the depth estimation and ST_LSD detection. Section 4 presents the multi-constraint geometric modeling, covering epipolar geometry-based candidate initialization, coplanarity constraint via depth reconstruction, local homography constraint, angular consistency and distance-ratio constraint. Section 5 describes the global line segment matching via multi-constraint optimization. Section 6 reports the experimental results and discussion. Finally, Section 7 concludes the paper.

## 2. Overview of the Proposed Method

**Figure 1:**
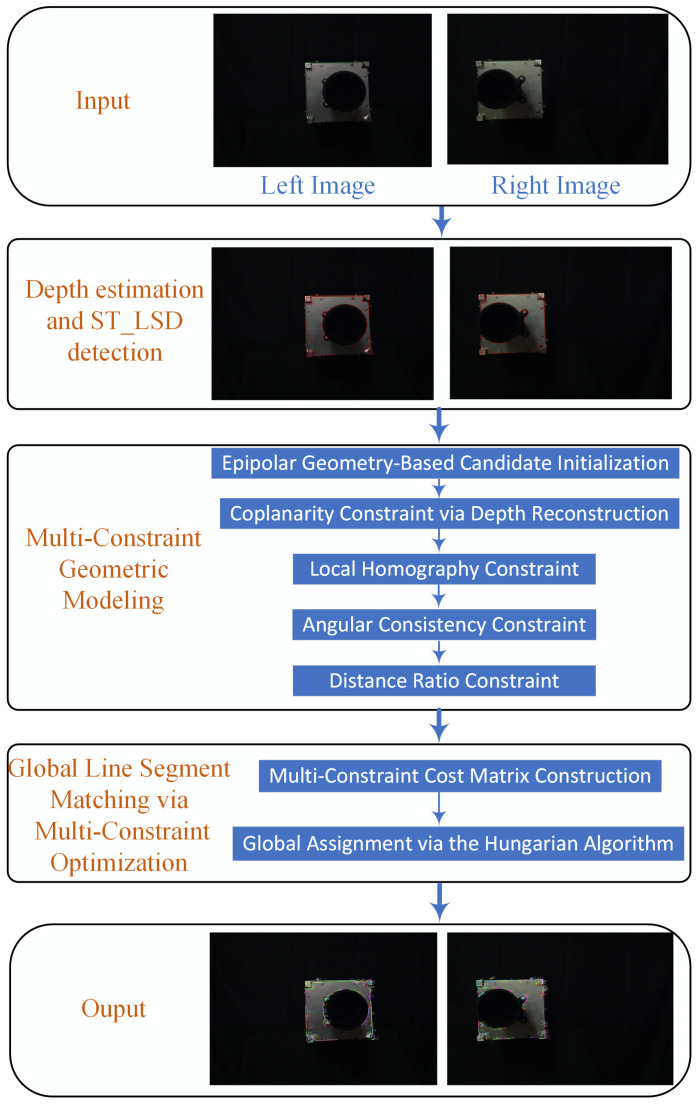
The flowchart of the proposed method.

The proposed framework targets robust stereo line segment matching under challenging spaceborne imaging conditions. As illustrated in Figure 1, the method proceeds through three sequential stages that are presented in Section 3, Section 4 and Section 5.

(1) **Depth estimation and ST_LSD detection**. Depth information is first estimated via sparse feature point correspondences to support subsequent verification of coplanarity constraints. Furthermore, the ST_LSD algorithm is introduced to detect line segments, providing a robust geometric basis for downstream matching tasks.
(2) **Multi-Constraint Geometric Modeling**. We formulate five complementary geometric constraints to evaluate the plausibility of each candidate correspondence: epipolar consistency, depth-aware coplanarity verification, local homography agreement, angular consistency, and distance-ratio preservation. These constraints are quantitatively modeled as normalized residuals, jointly encoding scene-wide 3D geometry and local 2D spatial relationships to provide a robust and complementary basis for evaluating candidate correspondences. The mathematical definitions of these constraints are provided in Section 4.
(3) **Global Line Segment Matching via Multi-Constraint Optimization**. All constraint responses are aggregated into a unified cost representation and cast as a Linear Assignment Problem (LAP). We solve the LAP via the Hungarian algorithm to ensure strict one-to-one global consistency, followed by pruning to remove unreliable correspondences; details of cost matrix construction and global assignment are provided in Section 5.2 and Section 5.3.

**Key Innovations.** Unlike conventional methods that implicitly assume coplanarity, we explicitly reconstruct the 3D coordinates of line segment endpoints via stereo triangulation and perform a geometric coplanarity check in 3D space. This physically grounded constraint enhances the reliability of correspondence validation.

Addressing the unique challenges of spaceborne imaging—such as low-texture surfaces, extreme illumination contrast, and significant sensor noise—we introduce the ST_LSD detector for reliable line segment extraction, thereby providing high-quality candidate features for subsequent matching.

We propose a unified multi-constraint global optimization framework that quantitatively integrates multiple complementary geometric constraints—including epipolar consistency, depth-aware coplanarity verification, angular consistency, and distance-ratio preservation—into a single normalized, adaptively weighted cost matrix. The resulting LAP is solved via the Hungarian algorithm, thus ensuring strict global geometric consistency in the matching results.

## 3. Depth Estimation and ST_LSD Detection

In on-orbit servicing missions, stereo images often suffer from strong illumination contrast, low-texture surfaces, and sensor-induced noise. Depth information, obtained from matched stereo keypoints via standard triangulation [44], supports subsequent geometric verification. For robust line detection under these challenging conditions, we adopt the ST_LSD algorithm [43], which employs a three-stage framework to extract reliable line segments for downstream matching.

(1) **Adaptive Bilateral Filtering:** The input image is first preprocessed using a scale-adaptive bilateral filter, which preserves structural edges while suppressing noise.
(2) **Improved Anchor Point Extraction:** An improved Otsu-based adaptive thresholding strategy is employed to segment bright–dark transition regions. Anchor points are extracted based on significant local gradient responses. These anchors are then proactively clustered into line segments by assessing the geometric alignment and directional coherence among adjacent points.
(3) **Line Validation and Aggregation:** Each candidate line segment is further validated based on the spatial density of its anchor points and the statistical distribution of their gradient magnitudes. Segments lacking sufficient support or exhibiting irregular gradient patterns are discarded to effectively suppress false positives.

Compared with standard line-segment detector (LSD) [45], the ST_LSD algorithm demonstrates superior robustness under uneven lighting and texture deficiency, producing stable line segments for the stereo image pair. These extracted segments form the foundation for subsequent geometric matching stages.

## 4. Multi-Constraint Geometric Modeling

To robustly validate line correspondences, we propose a coarse-to-fine progressive filtering framework incorporating multiple geometric constraints. Unlike conventional approaches that implicitly assume coplanarity, our method explicitly verifies the coplanarity of line segments using reconstructed depth information, followed by a series of constraint-based evaluations to refine the set of reliable line matches.

### 4.1. Epipolar Geometry-Based Candidate Initialization

To initialize geometrically consistent line segment correspondences, we employ a coarse-level pruning based on epipolar constraints. In stereo vision, the epipolar geometry defines the intrinsic projective relationship between corresponding points across a stereo pair.

**Figure 2:**
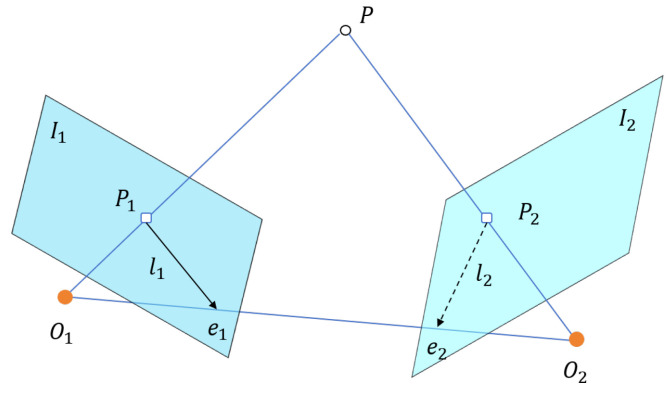
Illustration of the epipolar geometry.

As illustrated in Figure 2, which shows the epipolar geometry, given a matched pair of points $p_{1}$ and $p_{2}$ from the left and right images, the epipolar geometry imposes that $p_{2}$ should lie on the epipolar line $l_{2}=Fp_{1}$ in the right image, and vice versa for $p_{1}$ and $l_{1}=F^{⊤}p_{2}$ in the left image, where F is the fundamental matrix [44]. To achieve robust verification, we measure the symmetric epipolar distance (SED) as [46]

(1) $$D_{\mathrm{epi}}=\frac{1}{2}\left(\frac{|p_{2}^{⊤}Fp_{1}|}{∥Fp_{1}∥}+\frac{|p_{1}^{⊤}F^{⊤}p_{2}|}{∥F^{⊤}p_{2}∥}\right),$$

which equally considers the projection error from both image domains, mitigating bias from single-view estimation.

### 4.2. Coplanarity Constraint via Depth Reconstruction

While traditional stereo line segment matching methods often rely on epipolar constraints and simplistic geometric priors, they typically assume that corresponding line segments lie on the same 3D plane—a condition frequently violated in realistic spaceborne scenarios involving complex structural layouts and diverse viewing angles.

**Figure 3:**
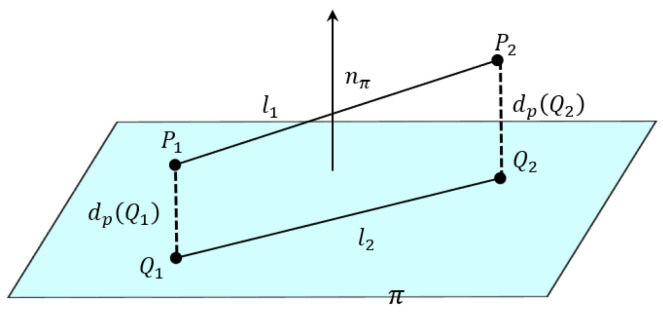
Illustration of the coplanarity constraint based on 3D line reconstruction.

In contrast, we propose a depth-aware coplanarity verification framework that moves beyond such assumptions by explicitly evaluating the spatial relationships of matched line pairs in 3D. Specifically, we reconstruct line segments in 3D space and assess whether they share a common supporting plane based on geometric consistency. An illustration of this constraint is provided in Figure 3.

Let two 3D line segments be denoted as $\ell_{1}=\left(P_{1},P_{2}\right)$ and $\ell_{2}=\left(Q_{1},Q_{2}\right)$, where $P_{i},Q_{i}\in R^{3}$ are the reconstructed endpoints in Euclidean coordinates. A supporting plane π is constructed using the three non-collinear points $P_{1}$, $P_{2}$, and $Q_{1}$. The unit normal vector of the plane is computed by [47,48]

(2) $$n_{\pi }=\frac{\left(P_{2}-P_{1}\right)\times \left(Q_{1}-P_{1}\right)}{∥\left(P_{2}-P_{1}\right)\times \left(Q_{1}-P_{1}\right)∥}.$$

Let $x\in R^{3}$ denote an arbitrary 3D point. The signed distance from x to the plane π is given by

(3) $$d_{\pi }\left(x\right)=\frac{{\left(x-P_{1}\right)}^{⊤}n_{\pi }}{∥n_{\pi }∥}.$$

To quantify the coplanarity of line $\ell_{2}$ with respect to π, we define the coplanarity residual as the average distance of its endpoints to the plane:

(4) $$D_{\mathrm{cop}}=\frac{1}{2}\left(\left|d_{\pi }\left(Q_{1}\right)\right|+\left|d_{\pi }\left(Q_{2}\right)\right|\right).$$

If $D_{\mathrm{cop}}<\varepsilon$, where ε is a predefined threshold reflecting tolerance to reconstruction noise (typically within 0.5–2.0 mm depending on stereo baseline and depth accuracy), the two line segments are considered coplanar. This physically grounded constraint enables the rejection of false matches caused by ambiguous textures, overlapping projections, or structural occlusions.

To ensure clarity and reproducibility, the detailed coplanarity verification procedure is summarized in Algorithm 1.
**Algorithm 1:** Depth-Guided Coplanarity Check
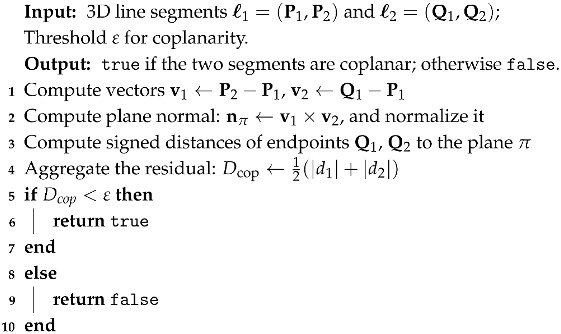


### 4.3. Local Homography Constraint

To further verify the geometric consistency of candidate line segments, we adopt the local homography constraint inspired by the GLSM framework. If two matched line segments are projections of the same physical 3D line lying on a locally planar surface, the spatial distribution of their nearby supporting points should approximately satisfy a projective transformation [31].

**Figure 4:**
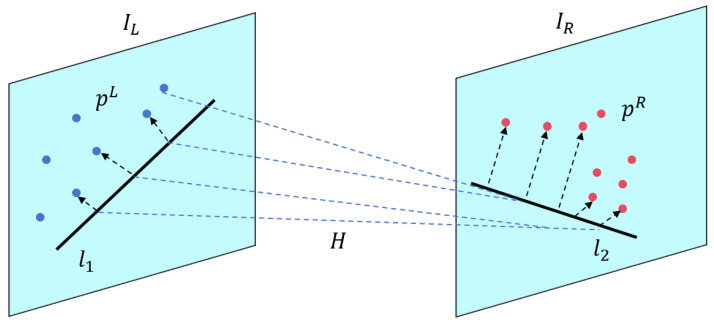
Illustration of the local homography constraint.

As illustrated in Figure 4, let $\ell_{1}$ and $\ell_{2}$ denote a matched line pair in the left image $I_{L}$ and right image $I_{R}$, respectively. Around each line, a set of neighboring supporting points is sampled (e.g., gradient-aligned or SURF matches), denoted as ${\left\{p_{i}^{L}\right\}}_{i=1}^{N}$ and ${\left\{p_{i}^{R}\right\}}_{i=1}^{N}$. Under the planarity assumption, these two neighborhoods are related by a homography matrix $H\in R^{3\times 3}$ [44]:

(5) $$p_{i}^{R}∼Hp_{i}^{L}.$$

The homography H is estimated using at least four matched point pairs, and the average reprojection error is computed as

(6) $$D_{\mathrm{hom}}=\frac{1}{N}\sum_{i=1}^{N}{∥p_{i}^{R}-{\hat{p}}_{i}^{R}∥}_{2},\;{\hat{p}}_{i}^{R}=\frac{Hp_{i}^{L}}{∥Hp_{i}^{L}∥},$$

where ${\hat{p}}_{i}^{R}$ is the projected location of $p_{i}^{L}$ under H. A small $D_{\mathrm{hom}}$ indicates that the neighborhood around $\ell_{1}$ can be accurately transformed to that around $\ell_{2}$, supporting the hypothesis that they are projections of the same 3D structure on a locally planar surface [31].

### 4.4. Angular Consistency Constraint

To further enhance matching precision, we introduce two geometric consistency constraints based on the structural invariance of line pairs: the angular constraint and the distance-ratio constraint. These constraints exploit the relative configuration of line segments and are particularly effective in resolving ambiguities caused by rotational transformations and viewpoint shifts.

Pose variation is common among non-cooperative space targets, leading to geometric deformations across stereo views. To address this, we incorporate an angular consistency constraint based on the principle that the relative angle between homologous lines remains approximately invariant under stereo projection.

Let $\ell_{i}$ and $\ell_{j}$ be two line segments in the left image, with corresponding candidates $\ell_{i}^{\prime }$ and $\ell_{j}^{\prime }$ in the right image. Denote their unit direction vectors as $d_{i}$, $d_{j}$, $d_{i}^{\prime }$, and $d_{j}^{\prime }$, respectively. The inter-line angles in each view are computed as [33]

(7) $$\theta_{ij}=\arccos\left(\frac{d_{i}^{⊤}d_{j}}{∥d_{i}∥\;∥d_{j}∥}\right),\;\theta_{ij}^{\prime }=\arccos\left(\frac{{d_{i}^{\prime }}^{⊤}d_{j}^{\prime }}{∥d_{i}^{\prime }∥\;∥d_{j}^{\prime }∥}\right)$$

The angular residual is then defined as the absolute difference between the original and matched angles:

(8) $$D_{\mathrm{angle}}=\left|\theta_{ij}-\theta_{ij}^{\prime }\right|$$

A small value of $D_{\mathrm{angle}}$ indicates that the orientation relationship between the line pair is preserved across views. Candidate matches are retained only if the residual satisfies $D_{\mathrm{angle}}<\tau_{\theta }$, where $\tau_{\theta }$ is a predefined angular tolerance threshold.

### 4.5. Distance-Ratio Constraint

In addition to enforcing orientation consistency, we impose a constraint on the relative spatial distribution of matched line segments. This constraint is based on the assumption that inter-line distances, expressed as ratios, are approximately preserved under perspective projection when the lines reside on the same physical surface.

Let $\ell_{i}$ and $\ell_{j}$ denote two line segments in the left image, and $\ell_{i}^{\prime }$ and $\ell_{j}^{\prime }$ their corresponding matches in the right image. We define the midpoints of these segments as $m_{i}$, $m_{j}$ in the left image and $m_{i}^{\prime }$, $m_{j}^{\prime }$ in the right image. The Euclidean distances between the midpoints are given by

(9) $$r_{ij}=∥m_{i}-m_{j}∥,\;r_{ij}^{\prime }=∥m_{i}^{\prime }-m_{j}^{\prime }∥$$

We then define the distance-ratio residual as

(10) $$D_{\mathrm{ratio}}=\left|\frac{r_{ij}}{r_{ij}^{\prime }}-1\right|$$

A small value of $D_{\mathrm{ratio}}$ indicates that the relative spacing between line segments is preserved across views, consistent with the expectation for coplanar structures under moderate perspective distortion. A match pair is retained only if the residual satisfies the constraint $D_{\mathrm{ratio}}<\tau_{r}$, where $\tau_{r}$ is an empirically determined threshold.

## 5. Global Line Segment Matching via Multi-Constraint Optimization

### 5.1. Overview of the Multi-Constraint Global Optimization Framework

Building upon the multiple geometric constraints introduced in Section 4, we reformulate stereo line segment matching as a multi-constraint global optimization problem. Instead of relying on a single cue or making isolated pairwise decisions, the proposed framework jointly exploits a set of complementary geometric cues—including epipolar consistency, depth-based coplanarity, local homography agreement, angular consistency, and distance-ratio preservation—to holistically assess the geometric plausibility of each candidate correspondence. This integration enforces geometric consistency across the entire set of detected line segments and suppresses mismatches that may arise from locally optimal but globally inconsistent decisions.

Within this unified formulation, the residuals from all constraints for each candidate pair are normalized and adaptively weighted to produce an aggregated cost that reflects overall geometric inconsistency. Aggregating these costs over all candidate pairs yields a complete cost matrix, which transforms the problem into a global one-to-one assignment task.

This optimization is accomplished in two sequential stages. First, Section 5.2 details the construction of the multi-constraint cost matrix by fusing all constraint residuals in a normalized, weighted manner. Second, Section 5.3 describes the solution of the resulting LAP via the Hungarian algorithm, followed by pruning to remove unreliable correspondences. This methodology provides a seamless transition from constraint-level modeling to global optimization, forming a mathematically consistent and robust framework for stereo line segment matching.

### 5.2. Multi-Constraint Cost Matrix Construction

In the first stage of the optimization, we construct a comprehensive cost matrix $C\in R^{M\times N}$, where *M* and *N* denote the numbers of line segments detected in the left and right images, respectively. Each element $C_{ij}$ encodes the aggregated geometric inconsistency between line segment $\ell_{i}$ in the left image and $\ell_{j}^{\prime }$ in the right image.

Five normalized geometric residuals are combined:

(1) $D_{\mathrm{epi}}$: Symmetric epipolar distance (Equation (1));
(2) $D_{\mathrm{cop}}$: Depth-based coplanarity residual (Equation (4));
(3) $D_{\mathrm{hom}}$: Local homography reprojection error (Equation (6));
(4) $D_{\mathrm{angle}}$: Angular inconsistency between line pairs (Equation (8));
(5) $D_{\mathrm{ratio}}$: Distance-ratio deviation (Equation (10)).

All residuals are independently normalized to [0,1] via min–max normalization over the candidate space, and then linearly combined using adaptive weights:

(11) $$C_{ij}=\alpha D_{\mathrm{epi}}^{\left(ij\right)}+\beta D_{\mathrm{cop}}^{\left(ij\right)}+\gamma D_{\mathrm{hom}}^{\left(ij\right)}+\delta D_{\mathrm{angle}}^{\left(ij\right)}+\epsilon D_{\mathrm{ratio}}^{\left(ij\right)}$$

subject to

(12) α+β+γ+δ+ϵ=1,α,β,γ,δ,ϵ≥0

The weights can be tuned empirically or optimized via cross-validation. In our implementation, (α,β,γ,δ,ϵ)=(0.3,0.25,0.05,0.2,0.2) achieves a balanced integration across constraints with different sensitivities.

### 5.3. Global Assignment via the Hungarian Algorithm

With the cost matrix C obtained in Section 5.2, the stereo line segment matching problem becomes an LAP, where the goal is to find a bijective mapping $\pi :\ell_{i}\to \ell_{\pi (i)}^{\prime }$ that minimizes the total matching cost [49]:

(13) $$\min_{\pi }\sum_{i=1}^{M}C_{i,\pi (i)}$$

We solve this optimization exactly using the Hungarian algorithm, which guarantees a globally optimal solution in polynomial time. To further suppress unreliable or ambiguous correspondences, a global matching threshold $\tau_{c}$ is applied so that only pairs with sufficiently low geometric inconsistency are retained:

(14) $$C_{ij}<\tau_{c}$$

This pruning ensures that the final correspondence set satisfies one-to-one matching and strict geometric consistency, thereby reducing over-assignment in low-texture or perspective-distorted regions.

## 6. Experiments and Discussion

This section presents a comprehensive and well-structured experimental evaluation of the proposed line segment matching method, including both quantitative and qualitative assessments. The performance of our approach is compared with three representative algorithms: LBD, LJL, and GLSM. Specifically, LBD is a classical line segment matching technique, LJL has been widely adopted in recent years as a baseline method, and GLSM is a well-recognized state-of-the-art approach. Through extensive analysis and comparative experiments, our method demonstrates superior accuracy and robustness across diverse scenarios. These findings suggest that the proposed approach offers a promising alternative for reliable and precise line segment matching.

### 6.1. Evaluation Metrics

We employ three standard metrics to assess line segment matching performance: precision (P), recall (R), and F-score (F), defined, respectively, as

(15) $$\begin{array}{cc} \mathrm{Precision} & =\frac{N_{\mathrm{correct}}}{N_{\mathrm{matched}}}, \end{array}$$

(16) $$\begin{array}{cc} \mathrm{Recall} & =\frac{N_{\mathrm{correct}}}{N_{\mathrm{gt}}}, \end{array}$$

(17) $$\begin{array}{cc} F\text{-}\mathrm{score} & =\frac{2\;\cdot \;P\;\cdot \;R}{P+R}. \end{array}$$

where $N_{\mathrm{correct}}$ is the number of correctly matched line pairs, $N_{\mathrm{matched}}$ is the total number of matches produced by the algorithm, and $N_{\mathrm{gt}}$ denotes the total number of ground-truth correspondences.

### 6.2. Dataset Description

To ensure a rigorous and fair evaluation of line segment matching algorithms, two types of stereo datasets are employed in this study: a self-built dataset acquired using a semi-physical simulation platform, and the publicly available Line Matching Benchmark dataset [50]. The combination of these datasets enables both task-specific validation under controlled conditions and comprehensive assessment under diverse real-world scenarios.

#### 6.2.1. Self-Built Stereo Line Segment Matching Dataset

**Figure 5:**
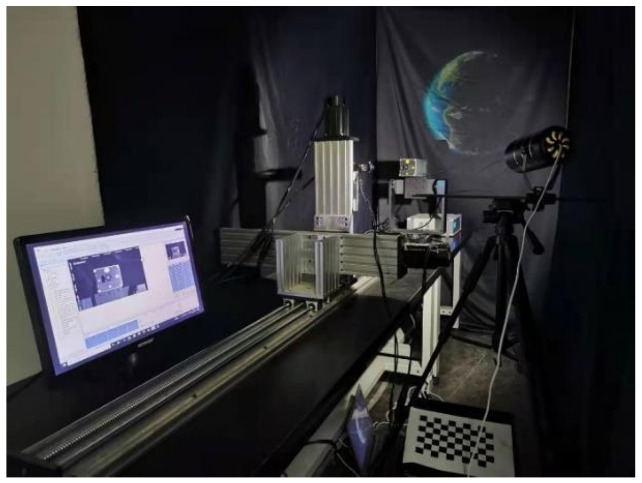
Illustration of the semi-physical simulation platform for dataset acquisition.

This study focuses on line segment matching algorithms for non-cooperative space targets, which require reliable experimental data for thorough validation. However, existing stereo datasets specifically designed for such targets are limited. To enable a more rigorous and effective performance analysis, we constructed a semi-physical simulation platform that emulates the key photometric attributes of the space environment, namely directional and high-contrast illumination, while allowing for controlled data acquisition. As illustrated in Figure 5, the experimental system consists of a three-degree-of-freedom linear rail, a three-axis turntable, a solar illumination simulator, a stereo camera setup with adjustable baseline, two physical CubeSat models, and a background panel representing Earth. The solar simulator emulates the lighting conditions in space, while the linear rail captures target images at varying distances and the turntable controls target orientation. This semi-physical platform enables the generation and acquisition of datasets tailored for the validation of stereo line segment matching algorithms.

To ensure experimental integrity and facilitate reproducibility, we summarize the key configurations of the illumination simulator and stereo imaging system in Table 1. The solar simulator provides controllable light intensity and incident angles, approximating the directional and high-contrast nature of real space illumination. Notably, the solar simulator employs the AM1.5G terrestrial standard, which in our setup serves as a controllable, high-intensity directional source. We do not claim spectral equivalence to the AM0 spectrum of outer space; rather, the simulator provides an operational approximation of the directional and high-contrast illumination conditions most relevant to the evaluation of line detection and matching in spaceborne imagery. The stereo cameras allow adjustable baselines and lens settings to simulate different onboard imaging configurations.

It is worth noting that the proposed semi-physical platform provides both controllability and representativeness. The solar illumination simulator reproduces high-contrast, directional lighting analogous to orbital conditions, while enabling systematic variations in intensity and incidence. Nonetheless, it cannot fully reproduce the AM0 spectral composition, long-term radiation effects, complex specular reflections, or large-scale background contributions from Earth albedo. To provide basic validation, we measured luminance statistics directly from the captured images; the mean $\mu_{Y}$, near-dark proportion $\gamma_{s}$, and near-saturated proportion $\gamma_{h}$ indicate that the generated illumination yields contrast and the shadowing characteristic of space-like conditions.

Regarding target diversity, two CubeSat models with distinct geometric complexity are currently employed: a simple cubic body and another with protruding panels and surface details. This setting ensures validation on both basic and moderately complex geometries. To further enhance generalization, additional satellite models with higher structural complexity (e.g., antennas, solar arrays) will be incorporated in future experiments. Overall, the semi-physical simulation platform enables the acquisition of datasets under controlled illumination and geometric conditions, meeting the requirements for stereo line segment matching of non-cooperative space targets.

**Figure 6:**
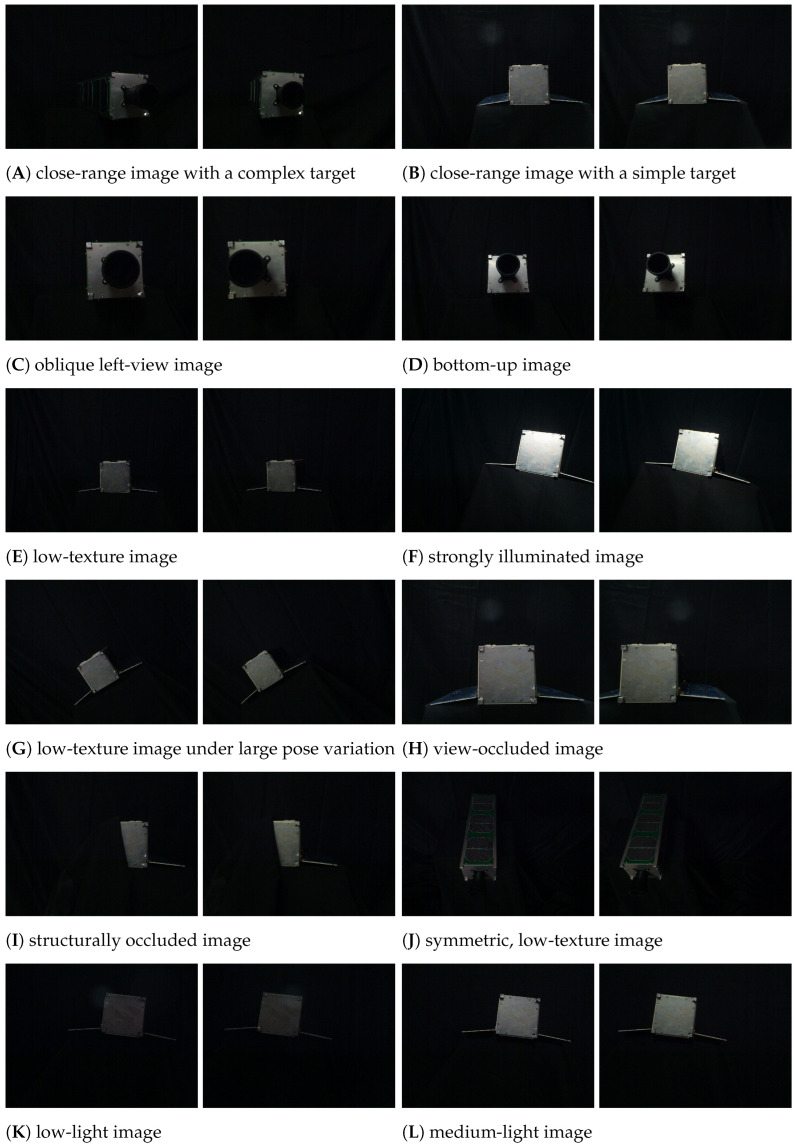
Twelve representative stereo image pairs used in matching evaluation.

Twelve representative stereo image pairs were selected to evaluate the performance of the proposed line segment matching algorithm, as illustrated in Figure 6A–L. The selected scenes include (A) close-range image with a complex target, (B) close-range image with a simple target, (C) oblique left-view image, (D) bottom-up image, (E) low-texture image, (F) strongly illuminated image, (G) low-texture image under large pose variation, (H) view-occluded image, (I) structurally occluded image, and (J) symmetric low-texture image, together with two additional illumination cases: (K) low-light image and (L) medium-light image.

The combination of subfigure (F), subfigure (K), and subfigure (L) provides three representative illumination levels, thereby extending the coverage of lighting variability in the dataset. To ensure reproducibility and avoid subjective categorization, illumination levels were quantitatively defined in the image domain based on 8-bit luminance statistics, including the global mean intensity $\mu_{Y}$, the proportions of near-dark ($\gamma_{s}=\mathrm{Pr}\left(Y\leq 5\right)$), and near-saturated pixels ($\gamma_{h}=\mathrm{Pr}\left(Y\geq 250\right)$). Similar luminance-based characterization strategies have been widely adopted in image quality assessment and illumination analysis literature [51,52], providing a consistent basis for operational definitions of illumination levels. The three cases yield $(\mu_{Y},\gamma_{s},\gamma_{h})=$ (21.96, 16.4%, 0.43%) for strongly illuminated image (subfigure (F)), (5.90, 75.0%, 0.0%) for low-light image (subfigure (K)), and (11.08, 43.4%, 0.0%) for medium-light image (subfigure (L)). These values confirm that the selected images cover a broad spectrum of illumination conditions in a scientifically consistent manner.

#### 6.2.2. Public Line Matching Benchmark Dataset

As no public dataset exists for line segment matching of non-cooperative space targets, we adopt the Line Matching Benchmark dataset [50], which has been widely used for line feature matching evaluation. It contains stereo pairs captured under diverse conditions (e.g., low-texture, scale changes, viewpoint variations), thereby complementing our self-built dataset.

**Figure 7:**
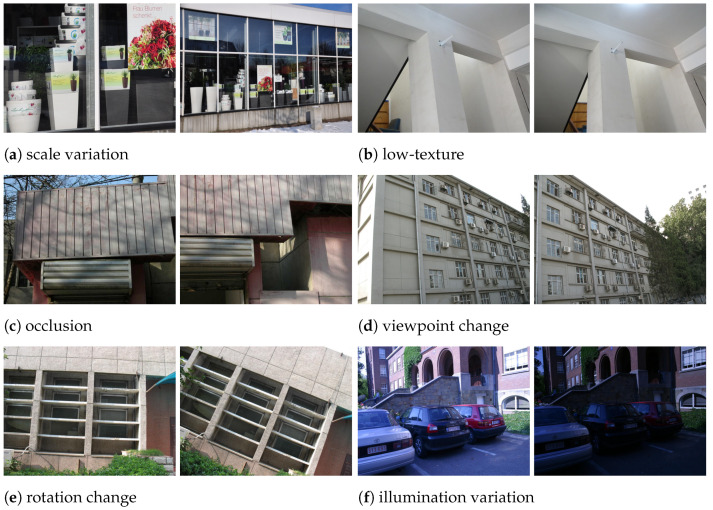
Six representative stereo image pairs selected from public benchmarks for line feature matching evaluation.

From this benchmark, six representative stereo pairs were selected to cover typical challenges in line segment matching, including (a) scale variation, (b) low-texture scenes, (c) occlusion, (d) viewpoint change, (e) rotation change, and (f) illumination variation (see Figure 7). These cases introduce additional difficulties such as geometric distortions, weak structural cues, and environmental variability, thus providing a rigorous supplement to the semi-physical dataset and enhancing the assessment of the proposed method’s generalization capability.

### 6.3. Parameter Settings

To ensure the effectiveness and fairness of the comparative evaluation, we detail the key parameter settings of three representative line segment matching algorithms: LBD, LJL, and GLSM. All methods are tested using either the original authors’ open-source implementations or re-implemented versions with parameter configurations faithfully reproduced from their publications.

For LBD, the Hamming distance threshold is set to 0.6, and the angular deviation threshold is set to 15°, following the original design to ensure both descriptor distinctiveness and geometric consistency. For LJL, the influential region width is set to 20 pixels and the descriptor radius to 10 pixels, which enables robust encoding of the local line context. For GLSM, which builds upon a graph-based coplanarity and plane hypothesis framework, the mapping error threshold is set to $T_{\mathrm{map}}=2$ pixels, and the overlapping rate threshold is set to $T_{\mathrm{ove}}=40\%$. These two parameters play a critical role in ensuring geometric consistency and enabling reliable line clustering under the coplanarity assumption. We use the publicly available implementations of the LJL (https://github.com/kailigo/LineSegmentMatching, accessed on 25 August 2025) and GLSM (https://skyearth.org/research/, accessed on 25 August 2025) algorithms, which are provided by the original authors. The parameter settings of our proposed method are empirically determined through extensive experiments. As summarized in Table 2, these parameters are relatively easy to tune and generalize well across different datasets.

### 6.4. Quantitative Evaluation

Building upon the evaluation metrics defined in Section 6.1, we now present a comprehensive quantitative analysis of the proposed method. The evaluation is conducted on two complementary datasets: (i) the self-built stereo line segment matching dataset tailored for non-cooperative space targets, and (ii) the widely adopted Line Matching Benchmark dataset [50]. These two datasets respectively emphasize application-specific validation and generalization ability, thereby ensuring a balanced and rigorous assessment.

#### 6.4.1. Evaluation on Self-Built Dataset

Table 3 presents the line segment matching performance of four representative methods across 12 stereo image pairs. As shown, the proposed method achieves the highest precision and F-score, surpassing the second-best GLSM by approximately 13% and 4%, respectively, while attaining a slightly lower recall. The superior precision and F-score are attributed to the effective exploitation of stereo geometry: depth information is computed to perform coplanarity verification, and a cascade of geometric constraints is applied in a coarse-to-fine manner. This hierarchical framework significantly improves the accuracy and robustness of line segment matching. However, the multi-constraint filtering also leads to a more conservative matching behavior, resulting in a moderate decrease in recall. Compared with GLSM, this difference can be explained from their algorithmic principles: GLSM leverages a line-point graph ranking strategy that retains a broader set of candidate correspondences, thereby favoring higher coverage of true matches and thus higher recall. In contrast, our method adopts stricter geometric consistency checks and performs one-to-one global optimization via the Hungarian algorithm. While this stringent design substantially suppresses false positives and ensures more reliable overall performance, it inevitably discards some borderline correct matches (e.g., weakly constrained or slightly noisy ones), which accounts for the marginally lower recall.

LJL ranks third in precision by leveraging junction-based geometric context to suppress false matches. However, its strict local structure assumptions result in the exclusion of many true matches, leading to the lowest recall among all methods. In contrast, the classical LBD method exhibits the lowest F-score overall. As it relies solely on binary descriptors and brute-force Hamming distance matching without any geometric validation, it lacks robustness to appearance variation and structural ambiguity—despite offering high computational efficiency.

**Figure 8:**
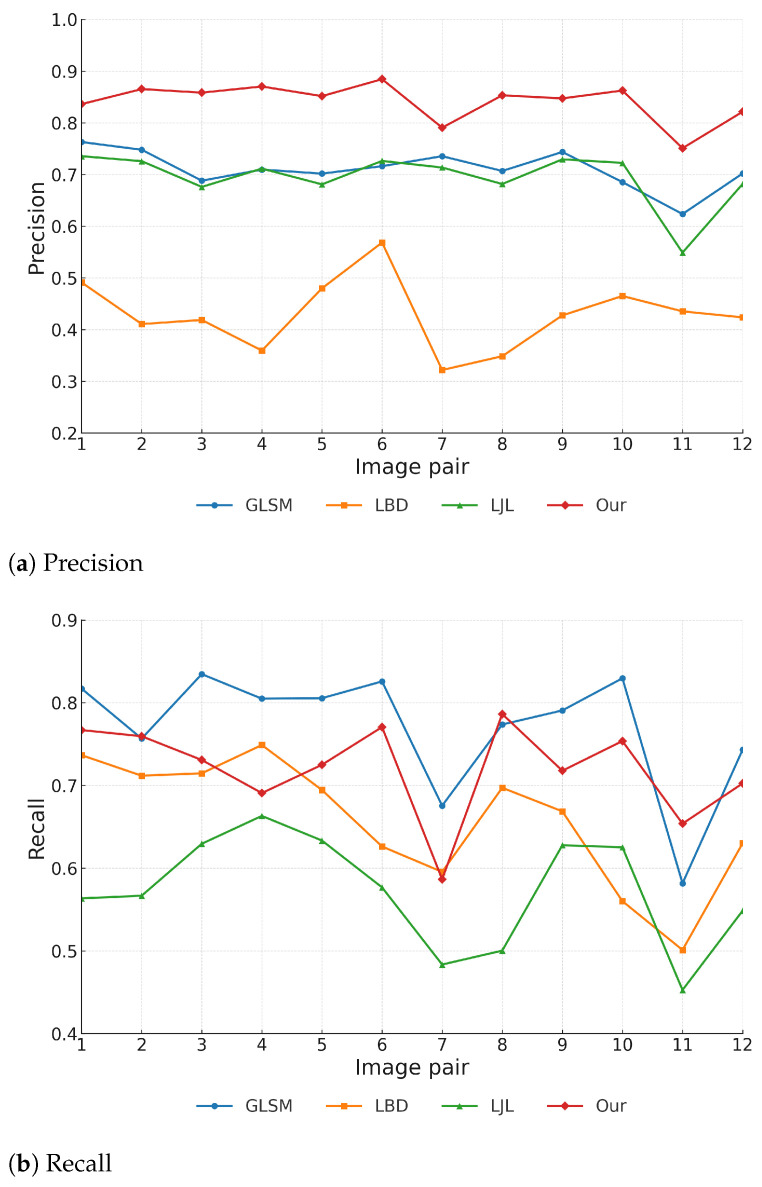

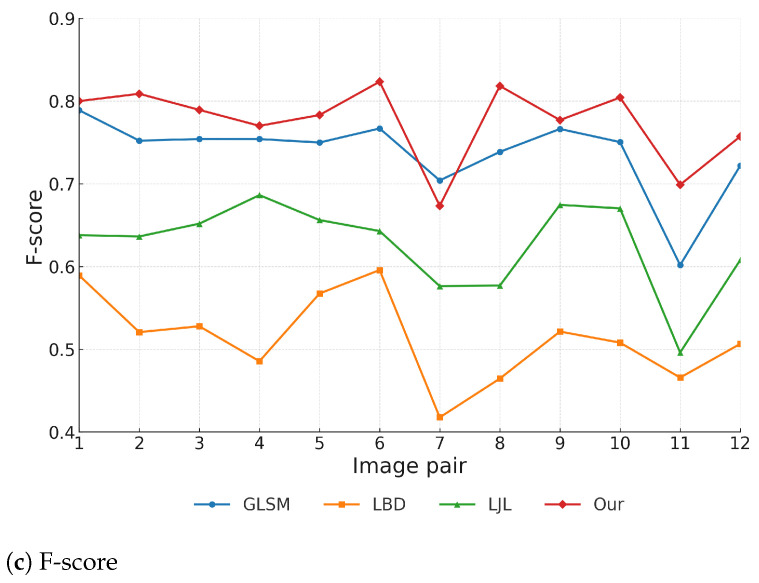
Quantitative evaluation results on 12 representative stereo image pairs.

Figure 8 presents the quantitative evaluation results of four line segment matching methods across 12 stereo image pairs. Our proposed method achieves consistently superior precision on all image pairs, maintaining a clear advantage over the other approaches. In contrast, LBD shows the weakest precision, ranking consistently last.

In terms of recall, GLSM generally achieves the highest values, likely due to its graph-based optimization framework that favors broader match coverage. Nevertheless, our method maintains competitive recall and remains among the top two methods across most image pairs, demonstrating strong stability and generalizability.

For the F-score, our method achieves the best overall performance, ranking first on the majority of image pairs. Although it falls slightly below GLSM in a few cases due to its stricter geometric filtering, the combination of high precision and competitive recall ensures superior robustness and accuracy under diverse stereo conditions.

#### 6.4.2. Evaluation on Public Benchmark

To further assess the robustness and generalization capability of the proposed method, we conducted experiments on the widely used Line Matching Benchmark dataset [50]. This benchmark provides stereo image pairs together with line segments extracted by LSD and ED detectors, along with manually verified correspondences. To ensure fairness and highlight the performance of matching strategies rather than detection quality, all competing methods were evaluated on the same ground-truth-aligned LSD detections. In particular, on the public benchmark dataset, our method is again compared with the representative baselines LBD [17], LJL [26], and GLSM [31], ensuring consistency with the evaluation on our self-built dataset.

Table 4 summarizes the quantitative results on six representative stereo pairs. The proposed method still achieves the best overall performance in terms of both precision and F-score, with precision in particular exceeding that of the second-best method, GLSM, by approximately 6.03%. While GLSM attains the highest recall (0.8474) owing to its graph-ranking strategy that favors broader match coverage, our approach demonstrates a more favorable balance between accuracy and robustness. In contrast, LBD shows the weakest performance, with both precision and recall slightly above 0.50, resulting in the lowest F-score overall. LJL achieves a relatively higher precision of about 0.71, but its recall remains limited (around 0.53), which constrains its overall F-score. These comparisons highlight that, unlike LBD and LJL, which struggle to maintain balanced performance, our method achieves a superior trade-off between precision and recall, thereby ensuring consistently higher matching reliability.

It is worth noting that, on the public dataset, the performance improvement of our method over the second-best GLSM is comparatively modest. This is mainly due to the different line detection strategies: in our self-built dataset, we employed ST_LSD to generate more complete and stable structural cues, whereas in the public benchmark, all methods are constrained to the same LSD detections. The absence of the enhanced detection module reduces the relative advantage of our pipeline. Nevertheless, the consistent superiority of our method across both datasets demonstrates its strong generalization capability and robustness against variations in scene complexity.

**Figure 9:**
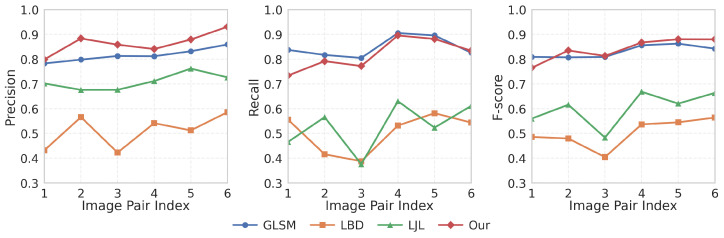
Quantitative evaluation results on six representative stereo pairs from the line matching benchmark.

Figure 9 presents the pairwise comparison of precision, recall, and F-score across six stereo pairs. Our method exhibits the most stable precision curve, consistently outperforming the baselines. For recall, GLSM leads slightly in several cases, yet our method remains highly competitive and never falls below the second-best. In terms of F-score, our approach achieves the top ranking in five out of six cases, highlighting its effectiveness in balancing precision and recall under diverse matching conditions. These complementary results indicate that the proposed method is effective not only in application-specific scenarios but also on widely used public benchmarks, which validates its generalization capability.

### 6.5. Qualitative Evaluation

Since the public dataset already provides manually validated ground-truth correspondences, we primarily focus on quantitative results for this benchmark, while the qualitative visualization is demonstrated on our self-built dataset to better highlight application-specific scenarios.

Notation for all qualitative figures: identical color and index across the left/right views denote one hypothesized correspondence; orange boxes mark representative regions discussed in the paper.

#### 6.5.1. Analysis Under Close-Range Complex Target

**Figure 10:**
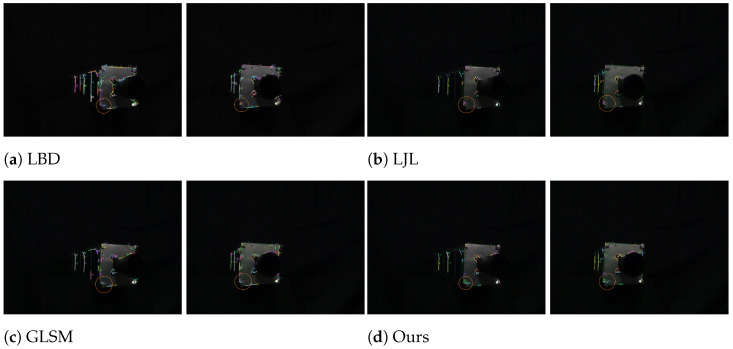
Qualitative results under close-range complex target. Orange boxes highlight regions of interest. LBD yields only incorrect pairs (#4, #38, #64, #67, #72); LJL finds one correct pair (#25); GLSM produces correct matches (#31, #37, #48) but also an error (#4); our method returns only correct correspondences (#23, #28, #34, #39).

Figure 10 shows the qualitative results under a close-range complex target, which has significant structural complexity and rich details at relatively short imaging distances, representing common challenging scenarios in orbital service tasks.

As shown in Figure 10a by the orange circular area, the LBD method detects and matches multiple line segment pairs, but exhibits poor geometric consistency and structural correspondence, mainly manifested as obvious deviations in spatial position, direction, and shape of the line segments between the left and right images.

Figure 10b presents the matching result of the LJL method, where the orange circular area indicates fewer matches, only matching the line segment pair No. 25, but with high matching accuracy, consistent with the characteristics of the LJL algorithm.

The orange circular area in Figure 10c shows that the GLSM method successfully matches line segment pairs No. 31, 37, and 48, but incorrectly matches the line segment No. 4 in the right image, indicating that GLSM has strong matching capability and high recall for close-range complex images, but reduced precision.

In the highlighted orange circular region of Figure 10d, our proposed method successfully matches four line segment pairs No. 23, 28, 34, and 39, achieving 4/4 correct correspondences. In comparison, GLSM obtains 3/4, LJL 1/1, and LBD 0/5. Beyond achieving the highest number of correct correspondences, our method also preserves geometric accuracy, directional consistency, and structural coherence across matches. These results demonstrate the superior reliability of the proposed framework in handling close-range complex targets, where cluttered structures and dense edges pose significant challenges for conventional approaches.

#### 6.5.2. Analysis Under Strong Illumination

**Figure 11:**
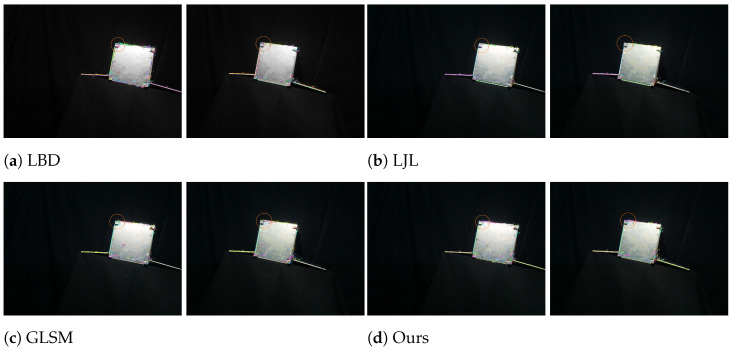
Qualitative results under strong illumination. Orange boxes indicate analyzed regions. LBD has a few correct matches (#41, #78) but many errors (#34, #48, #68, #70, #71, #76, #94); LJL conservatively matches one pair (#11); GLSM mixes correct pairs (#4, #19, #39) with mismatches (#1, #36, #22); our method identifies correct pairs (#3, #11, #19, #38) with only one error (#22).

Figure 11 shows the qualitative results under strong illumination. In spatial environments, strong illumination from direct sunlight and reflections often causes overexposure, texture loss, and edge blurring, posing significant challenges for line segment matching algorithms.

As shown in the orange circular area of Figure 11a, the LBD method detects numerous line segments in overexposed areas; however, the lack of discriminative textures and contrast produces many incorrect matches.

In contrast, the LJL method, as shown in the orange circular area of Figure 11b, only matches the line segment pair No. 11, demonstrating a conservative strategy that prioritizes match reliability over completeness.

The orange circular area in Figure 11c shows that GLSM correctly matches line segment pairs No. 4, 19, and 39, but erroneously matches No. 1 and 36 in the left image with No. 22 in the right image, which are not true correspondences.

In the highlighted orange circular region of Figure 11d, our proposed method correctly matches four line segment pairs No. 3, 11, 19, and 38, with only one error at No. 22. This corresponds to 4/5 correct correspondences, while GLSM achieves 3/6, LJL 1/1, and LBD only 2/9. In addition to maintaining the highest precision, our framework preserves geometric consistency and directional stability across matches. These results demonstrate that the proposed method remains robust and reliable for correspondence estimation under strong illumination variations.

#### 6.5.3. Analysis Under Symmetric and Low-Texture Scenario

**Figure 12:**
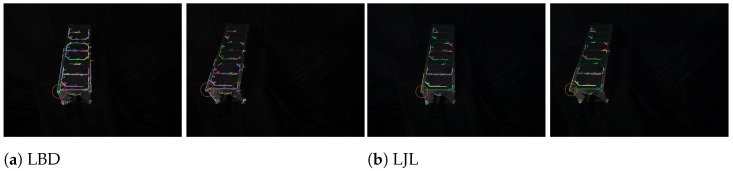

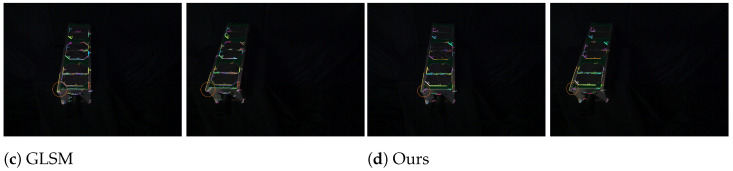
Qualitative results under symmetric and low-texture scenario. Orange boxes highlight ambiguous regions. LBD finds one correct pair (#81) but several mismatches (#21, #30, #35, #36, #50, #62); LJL detects one correct pair (#9); GLSM correctly matches (#8, #45) but also errors (#3, #13, #17, #29); our method produces only correct correspondences (#11, #25).

Figure 12 shows the qualitative results under symmetric and low-texture scenario. This scenario lacks distinctive local texture, which often results in ambiguous correspondences for matching algorithms. Furthermore, strong geometric symmetry can lead to mismatches in repetitive or visually similar regions.

As shown in the orange circular region of Figure 12a, the LBD method produces multiple segment matches, but only the pair No. 81 is correctly matched.

In contrast, Figure 12b shows that the LJL method successfully matches segment pair No. 9 in the orange circular region, consistently adhering to its conservative strategy that emphasizes matching precision.

Unlike the results in Figure 10c and Figure 11c, the GLSM method performs poorly in this scenario, as shown in Figure 12c. Although it correctly matches segment pairs No. 8 and 45, it also produces incorrect matches including segment pairs No. 3, 13, 17, and 29.

In the highlighted orange circular region of Figure 12d, our proposed method correctly matches two line segment pairs No. 11 and 25 with no mismatches. This corresponds to 2/2 correct correspondences, compared with GLSM (2/6), LJL (1/1), and LBD (1/7). In addition to achieving perfect accuracy within the region of interest, our framework effectively suppresses mismatches and preserves structural consistency. These results demonstrate that the proposed method maintains reliable performance even under the challenging conditions of symmetry and weak texture.

### 6.6. Runtime Analysis and Computational Complexity

In on-orbit servicing tasks, line segment matching algorithms must achieve high accuracy while maintaining low computational complexity, in order to meet the stringent real-time constraints and limited onboard resources of satellite systems. Excessive computational cost can lead to processing delays, increased power consumption, and even mission failure. Therefore, a comparative analysis of algorithmic complexity is essential.

**Experimental environment.** To ensure fairness, all methods were executed under identical hardware and software conditions on a Windows 10 laptop equipped with an Intel Core i7-8750H CPU (2.20 GHz) and 16 GB RAM. The LBD, LJL, and the proposed method were implemented in Visual C++, whereas GLSM adopted the hybrid Matlab–C++ implementation provided by the original authors. Each stereo pair was processed ten times independently, and the averaged runtime was reported to mitigate fluctuations from background processes and to ensure statistical robustness.

**Figure 13:**
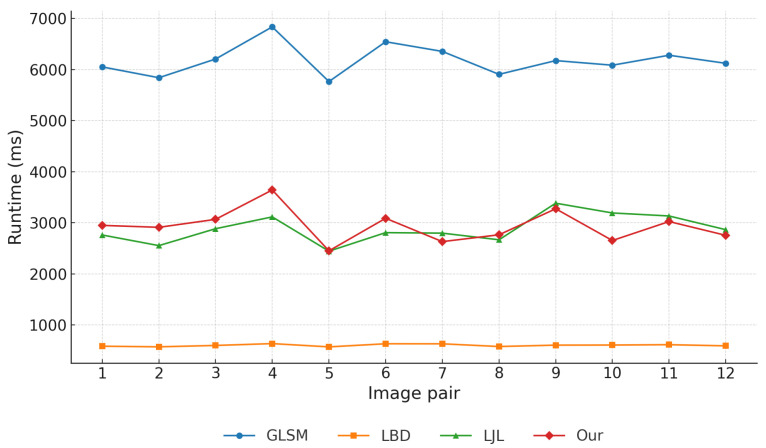
Runtime comparison of four line segment matching methods evaluated on 12 stereo image pairs.

**Overall runtime comparison.** Figure 13 shows the runtime of four representative methods across twelve stereo image pairs. LBD achieved the fastest runtime (∼600–700 ms), reflecting its lightweight design but sacrificing geometric robustness. LJL required significantly longer runtimes (2500–3300 ms), trading higher computational cost for stronger consistency. The proposed method achieved comparable runtimes to LJL (typically 2500–3000 ms), while GLSM was the slowest (∼2 × LJL), primarily due to its iterative random walk optimization and line-point graph construction. To facilitate deeper analysis, a representative runtime of 3000 ms is adopted for the proposed method.

**Module-wise runtime breakdown.** To better reveal computational characteristics, the runtime of the proposed method was decomposed into five key stages: (1) Depth estimation (37%, ∼1050 ms); (2) Line detection (25%, ∼750 ms); (3) Multi-constraint geometric modeling (18%, ∼600 ms); (4) Cost matrix construction (8%, ∼240 ms); (5) Hungarian optimization (12%, ∼360 ms).

**Figure 14:**
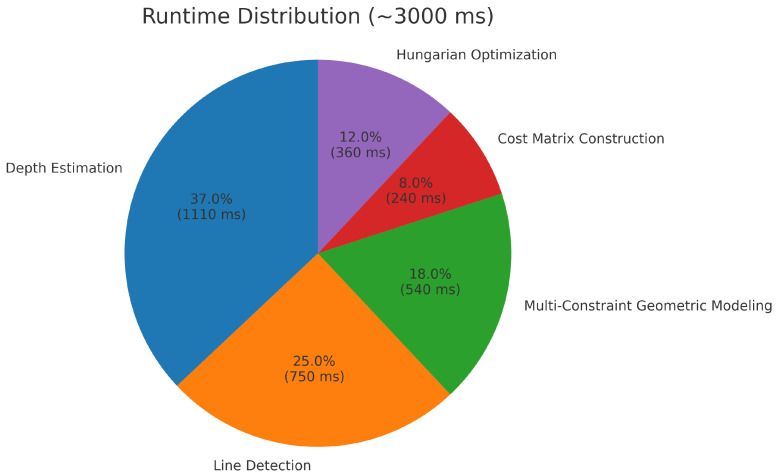
Module-wise runtime distribution of the proposed method (reference: 3000 ms total).

The breakdown is illustrated in Figure 14. Depth estimation and line detection dominate the runtime due to the large number of pixel- and gradient-level computations. The multi-constraint geometric modeling step introduces moderate overhead, since only progressively reduced candidate pairs are processed by the coplanarity, homography, angular, and distance-ratio constraints. In contrast, cost matrix construction and the Hungarian assignment remain relatively lightweight, as the number of surviving pairs before global optimization is already limited by the preceding filtering strategy. Nevertheless, Hungarian optimization is still retained as a necessary global step, enforcing one-to-one consistency beyond what local thresholds alone could achieve.

**Implications and discussion.** This analysis highlights that the primary bottlenecks lie in depth estimation and line detection, while subsequent geometric reasoning and global assignment consume smaller fractions of the total runtime. The decomposition also suggests clear optimization directions: GPU-based or parallelized depth estimation and SIMD-optimized line detection could substantially reduce runtime, thereby enhancing real-time applicability for on-orbit deployment. At the same time, the inclusion of Hungarian optimization, despite its moderate cost, ensures robust global matching, striking a balance between computational efficiency and geometric consistency.

In summary, based on extensive quantitative and qualitative evaluations as well as complexity comparisons, the proposed line segment matching method demonstrates excellent overall performance across multiple experimental scenarios. Even in challenging stereo images with structural symmetry, weak textures, or strong illumination, it maintains high matching accuracy and geometric consistency. Additionally, the multi-constraint progressive screening strategy effectively reduces computational overhead, achieving a favorable balance between matching accuracy and computational efficiency. To ensure robustness in practice, where imperfect rectification or front-end sparsity may occur (e.g., SURF yields too few matches or ST_LSD misses critical segments), the pipeline degrades safely; depth-dependent checks are bypassed, depth-free geometric constraints (epipolar, local homography, angular, and distance-ratio) and confidence-aware Hungarian assignments are retained, and no-match outputs are allowed when evidence is insufficient—prioritizing precision over recall. The experimental results consistently validate the robustness and effectiveness of the proposed method, highlighting its potential for deployment in resource-constrained applications such as on-orbit servicing.

## 7. Conclusions

In this work, we propose a robust and efficient line segment matching algorithm tailored for challenging stereo vision scenarios, particularly in space-based applications. Building upon a unified cost matrix that encodes multiple geometric constraints in a coarse-to-fine manner, our method achieves globally consistent and accurate matching through Hungarian optimization. Comprehensive evaluations across diverse and structurally complex stereo image pairs demonstrate that our approach achieves superior robustness, accuracy, and computational efficiency compared to several advanced baseline methods. A practical limitation is its reliance on reliable front-end detections (line segments and interest points); insufficient or unstable detections can diminish geometric discriminability and reduce recall. Future research will focus on extending the proposed matching framework to enable stereo-based pose estimation of non-cooperative space targets, further advancing its applicability in space perception and on-orbit autonomous operations. 

## Figures and Tables

**Table 1:** Essential configurations of the illumination simulator and stereo camera.

Category	Parameter	Value
Illumination Simulator	Standard	AM1.5G (Grade A)
	Spectral range	250–1100 nm
	Spot size	10 × 10–100 × 100 mm (square, adjustable)
	Irradiance uniformity	<5% (optionally ≤2%)
	Irradiance intensity	1000–50,000 mW/cm^2^
Stereo Camera System	Model	PXYZ-S-AR135-030T160
	Baseline	9.1 cm (adjustable)
	Lens focal length	3.4 mm (standard)
	Field of view	71° (horizontal)
	Resolution	2560 × 960
	Interface	USB 3.0 (UVC)

**Table 2:** The parameters used in our proposed method.

Step	Description	Symbol	Value
Section 4.1	Epipolar error threshold	$T_{\mathrm{epi}}$	3.0 pixels
Section 4.2	Coplanarity residual threshold	$T_{\mathrm{cop}}$	0.1
Section 4.3	Homography reprojection error	$T_{\mathrm{hom}}$	5.0 pixels
Section 4.4	Angular consistency threshold	$T_{\mathrm{ang}}$	π/6 (30°)
Section 4.5	Distance-ratio threshold	$T_{\mathrm{dis}}$	0.3

**Table 3:** Performance comparison across methods on our stereo image dataset.

Method	Precision	Recall	F-Score
LBD	0.4291	0.6571	0.5143
LJL	0.6943	0.5726	0.6262
GLSM	0.7101	0.7699	0.7375
Ours	0.8409	0.7204	0.7754

**Table 4:** Performance comparison across methods on the Line Matching Benchmark dataset.

Method	Precision	Recall	F-Score
LBD	0.5098	0.5026	0.5023
LJL	0.7087	0.5284	0.6017
GLSM	0.8158	0.8474	0.8308
Ours	0.8650	0.8179	0.8398

## Data Availability

The data underlying the results presented in this paper, which were collected in X.Z.’s laboratory, are not publicly available at this time but may be obtained from the author upon reasonable request.
